# Adolescents Hospitalized in an Acute Psychiatric Ward: The Difference between Males and Females in the Pre- and Pandemic/Post-Pandemic Periods

**DOI:** 10.3390/jcm13164658

**Published:** 2024-08-08

**Authors:** Rosaria Di Lorenzo, Pietro Bonasegla, Alice Bardelli Canzio, Martina Morgante, Sergio Rovesti, Paola Ferri

**Affiliations:** 1 Mental Health Department and Drug Abuse, AUSL-Modena, 41121 Modena, Italy; m.morgante@ausl.mo.it; 2 School of Specialization in Psychiatry, University of Modena and Reggio Emilia, 41121 Modena, Italy; pietro.bonasegla@hotmail.com; 3 Nursing Programme, University of Modena and Reggio Emilia, 41125 Modena, Italy; 224591@studenti.unimore.it; 4 Department of Biomedical, Metabolic and Neural Sciences, University of Modena and Reggio Emilia, 41125 Modena, Italy; sergio.rovesti@unimore.it (S.R.); paola.ferri@unimore.it (P.F.)

**Keywords:** impact of the pandemic, adolescent emotional disorders, aggressive and suicidal behavior

## Abstract

(1) **Background:** The pandemic lowered by 10% the psychological wellness among adolescents worldwide. (2) **Methods:** This observational retrospective study compared the demographic and clinical variables of male and female adolescents hospitalized in an acute psychiatric ward during the pre-pandemic, from 1 July 2017 to 28 February 2020, and the pandemic/post-pandemic, from 1 March 2020 to 30 June 2023. (3) **Results:** In total, 153 adolescents of 15.8 years on average (±1.14 DS) were more frequently hospitalized (*n* = 131, 54.4%) in the pre-pandemic than in the pandemic/post-pandemic (*n* = 110, 45.6%), but female hospitalizations increased in the post-pandemic more than male hospitalizations (62.9% vs. 37.1%) (Pearson Chi2 = 8.54, *p* = 0.003); in the pandemic/post-pandemic, we reported increased aggressive behavior and schizophrenia spectrum and emotional disorders in males, whereas in females, depressive and emotional disorders were prevalent; more adolescents previously treated in Child Mental Health Services were hospitalized in the pandemic/post-pandemic period; and males hospitalized in the study period reported higher Health of the Nation Outcome Scales for Children and Adolescents (HoNOSCA) scores. (4) **Conclusions:** In the pandemic/post-pandemic, females more frequently required hospitalizations for depressive behavior and males for aggressive behavior and schizophrenia spectrum disorders, whereas, in both males and females, hospitalizations due to emotional disorders increased. Our results suggest the need for mental health prevention in adolescents, who represent the most vulnerable population in the case of disaster.

## 1. Introduction

The number of adolescents suffering from psychiatric disorders has been constantly increasing over the last ten years, suggesting a real health emergency, which worsened during the pandemic [1]. According to the World Health Organization (WHO), the pandemic has worsened the mental health of minors worldwide by 10% [2]. Suicidal tendencies, self-harming behavior, and eating disorders [3] have all increased among adolescents [4].

### 1.1. The COVID-19 Pandemic and Adolescent Mental Health

The so-called “lockdown”, a period of restrictions and social isolation aimed at limiting the spread of the virus, influenced many aspects of daily life and was a particularly traumatic factor during the pandemic. People, especially teenagers, were forced to stay at home even to work and study (“smart-working” and “online teaching”), maintaining relationships only through social networking. During the lockdown, students experienced negative eating behaviors, altered sleeping patterns, and decreased physical activity during school closures [5]. The pandemic was a period of great worldwide stress which represented a risk factor for new outbreaks and complications of previous pathological conditions and predispositions [6,7] due to social isolation and other reasons strictly related to the outbreak. Anxiety stressors were represented by both the fear of losing loved ones because of the illness and the sense of guilt in case of being positive for COVID-19 infection [6]. Another consequence of prolonged social isolation for many young people was the loss of opportunities to develop important social skills. Research has shown that isolation during the pandemic may have increased the risk of social anxiety, resulting in many teens expressing fears about returning to in-person activities [8]. Furthermore, a SARS-CoV-2 infection, although usually associated with a mild form of COVID-19 among children, may have represented a disturbing and stressful experience due to potential isolation, stigmatization, or school disruption [9]. In particular, for today’s adolescents, who live in a generally overprotective social context, the reaction to the Coronavirus emergency has represented an unknown to which one could only respond after the fact [10,11].

### 1.2. Symptoms and Altered Behaviors in Adolescents during COVID-19

A Chinese survey from 2020 underscored that 37.4% of adolescents displayed anxiety disorders and 43.7% displayed symptoms of depression [12]. In Italy, a similar investigation reported an increase in post-traumatic symptoms associated with thought disorders and dissociative phenomena [13].

A study provided evidence of an increasing rate of suicide or suicidal thoughts associated with school closure and/or online learning [14]. Staying at home and the restrictions imposed by the lockdown have brought not only health but also personal crisis conditions, as evidenced by the many suicide attempts among students in China following school closures [12] and as reported by a recent systematic review [15]. The pandemic also represented a risk factor for non-destructive self-harm in under 18s, as documented by recent research in which the pandemic represented an enhanced stress factor on the continuation and development of Non-Suicidal Self-Injury (NSSI) among vulnerable adolescents [16].

In particular, an increasing suicide rate in populations younger than 20 years was observed in Japan in the second wave of the pandemic, immediately after school closures and some months after the lockdown (Incidence Rate Ratio—IRR 1.49; 95%CI 1.11–2.0 in September 2020 compared to the pre-pandemic period) [17].

In many cases, insomnia has been recognized as a pandemic stress-related issue [18]. In an Italian study, two independent large samples of adolescents aged 13–17 years were recruited at two pandemic time points: 1146 adolescents at time 1 (T1: April 2020) and 1406 at time 2 (T2: April 2021). Symptoms such as insomnia, altered sleep pattern and chronotype, psychological distress, and emotional dysregulation were collected. The prevalence of insomnia was 12.13% at T1 and 23.19% at T2 [19]. Furthermore, high levels of poor sleep habits (going to bed late, poor sleep hygiene, and the use of electronic devices before bed) were detected at both time points [20].

Systematic reviews conducted during the first year of the pandemic found a decrease in adolescents’ health-related quality of life (HRQoL) and mental health, with a worsening trend over time [21,22]. More recent studies have shown a stabilization or even a slight improvement in the well-being of adolescents two years after the pandemic outbreak [23,24]. This could be linked both to the development of strategies to deal with the pandemic situation and to the lifting of health measure restrictions [25].

The aim of the current study was to analyze the hospitalization of adolescents in an acute psychiatric ward during the last 6 years, comparing demographic and clinical variables in order to investigate changes in psychiatric diagnoses, hospitalization rates, and clinical outcomes, and how these changes differ between sexes across the pre- and the pandemic/post-pandemic periods.

## 2. Materials and Methods

This retrospective observational study involved data that concerned both the clinical and family aspects of participants under the age of 18 hospitalized in the Service for Psychiatric Diagnosis and Care (SPDC) of the Modena AUSL, located in the General Hospital of Baggiovara (Modena, Italy). The SPDC ward has 17 beds, 2 of which are dedicated to adolescents aged between 14 and 17 and which are located in a special section separate from the adult area, for a catchment area of approximately 700,000 inhabitants.

The Child and Adolescent Neuropsychiatry Service (CANPS), which collaborates with the psychiatrists of the SPDC department, includes eleven outpatient centers in the province of Modena, a center for attention deficit hyperactivity disorder, a center for eating disorders, and the residence “The Medlar” in a private but accredited nursing home exclusively for minors.

The study sample comprised 153 adolescents (under 18 years of age) who were admitted to the SPDC department (a total of 241 hospitalizations) over the study period (1 July 2017 through 30 June 2023). The following variables were collected: age, sex, previous psychiatric treatment, previous hospitalizations in SPDC, previous psychiatric treatment period, substance use, treatment adherence, clinical reason for hospitalization, hospitalization regime, duration of hospitalization, season of hospitalizations, psychiatric discharge diagnosis according to Classification of Diseases—9th revision—Clinical Modification (ICD-9-CM), and Health of the Nation Outcome Scales for Children and Adolescents (HoNOSCA) score.

### 2.1. Study Procedure

The sample of hospitalizations of patients under 18 years of age in the SPDC of the AUSL-Modena from 1 July 2017 to 30 June 2023 was divided into two periods: the pre-pandemic period, from 1 July 2017 to 28 February 2020, and the period following the COVID-19 outbreak from 1 March 2020 to 30 June 2023, called the pandemic/post-pandemic period.

The demographic and clinical variables relating to patients hospitalized in the two periods were compared and statistically analyzed. Furthermore, the sample of hospitalized adolescents was divided on the basis of sex, and the variables of the two groups were compared and statistically analyzed. The data were collected using two information platforms in use in the SPDC and in the Child and Adolescent Neuropsychiatry Service (CANPS), which collect demographic, clinical, and organizational information about patients and health interventions for two different information systems accessible only to healthcare workers. In particular, data were collected from the discharge letters available in the information system database used at the SPDC. The sample size was determined by identifying all cases of adolescent hospitalizations that met the inclusion criteria during the study period. No formal sample size calculation was necessary due to the complete inclusion of all eligible cases.

The collected data were anonymized by attributing a progressive alphanumeric code to each selected patient and their variables, successively reported in an Excel file and a STATA file for statistical analysis. The head of this study and his collaborators had access to the data, bound by the obligation of confidentiality and processing of the data in accordance with current regulations.

This study was approved by local Ethical Committee (Protocol: 0010824/23; 5 April 2023) and was authorized by the Direction of AUSL-Modena (Protocol 987; 27 April 2023).

### 2.2. Statistical Analysis

Descriptive statistical analyses of the sample were carried out: mean and standard deviations for all continuous variables; Student’s *t* tests for comparing means in a normally distributed sample evaluated by means of Skewness and kurtosis tests for normality, or Kruskal–Wallis for non-normal distribution; percentages for all categorical variables; and Pearson chi-squared and Fisher exact tests for percentage comparison and the analysis of Standardized Residuals (SR > or <2, *p* < 0.05). Two multiple linear regressions (stepwise, forward, and backward model) were applied between the independent variables represented by all the selected variables, as were two dependent variables, “duration of hospitalization” and “HoNOSCA score”, in order to explore factors influencing hospitalization length and severity of psychiatric conditions.

The probability significance level was set at *p* < 0.05.

The data were analyzed using STATA12 (Stata Corp., College Station, TX, USA, 2011).

## 3. Results

The study sample consisted of 153 adolescents with a mean age of 15.8 years (±1.14 SD) who underwent a total of 241 hospitalizations in the study period:

- In total, 110 hospitalizations (45.6%) in the pre-pandemic period (1 July 2017–28 February 2020);
- In total, 131 hospitalizations (54.4%) in the post-pandemic period (1 March 2020–30 June 2023).

### 3.1. Adolescent Hospitalizations in the Pre- and Pandemic/Post-Pandemic Periods

**Figure 1:**
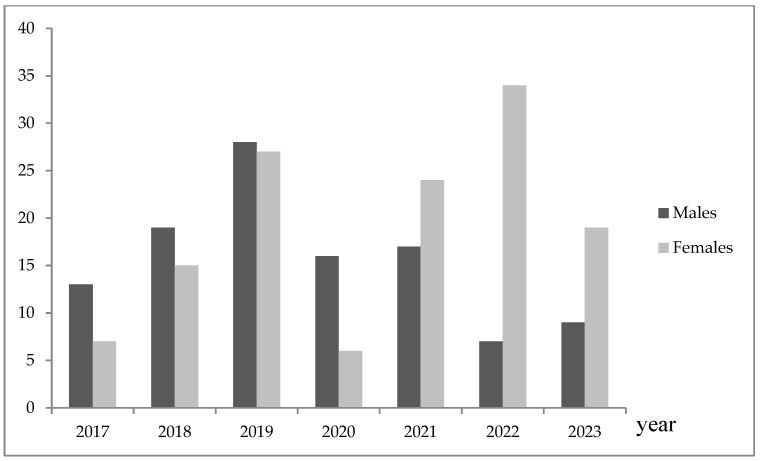
Male and female adolescent hospitalizations per year in the study period.

The adolescents in our sample (*n* = 153) were more frequently hospitalized in the pre-pandemic (*n* = 131, 54.4%) than in the pandemic/post-pandemic (*n* = 110, 45.6%). As shown in Figure 1, the frequency of adolescent hospitalizations was statistically significantly different in males and females in the whole study period (Pearson Chi2 = 27.01, *p* = 0.000). In the pre-pandemic period, males were more frequently hospitalized, whereas female adolescents were more often hospitalized in the pandemic/post-pandemic compared to the previous period (Pearson Chi2 = 8.54; *p* = 0.003) (Table 1, Figure 1). In particular, male hospitalizations were prevalent in 2017 (SR = 2.35, *p* < 0.05) and 2020 (SR = 2.88, *p* < 0.05), and female hospitalization was prevalent in 2022 (SR = 3.95, *p* < 0.05).

In Table 1, demographic and clinical variables related to adolescents hospitalized in the SPDC during the pre- and pandemic/post-pandemic periods are shown. The statistically significant differences between the two periods are represented by the following:

- The mean age of adolescents was lower (15.63 ± 1.18) during the pandemic/post-pandemic period (Chi2 = 3.915, Kruskal–Wallis test, *p* = 0.0478);
- More adolescents treated at CANPS were hospitalized in the pandemic/post-pandemic period (SR = 4.52, *p* < 0.05), and more adolescents without any previous psychiatric treatment were hospitalized in the pre-pandemic (SR = 4.38, *p* < 0.05) (Pearson Chi2 = 21.21, *p* = 0.000; Fisher’s exact = 0.000);
- More adolescents used substances in the pandemic/post pandemic period in comparison with the previous period (Pearson Chi2 = 5.36, *p* = 0.005), especially cannabis.

Regarding the clinical variables related to the psychiatric hospitalizations of adolescents in the SPDC during the pre- and pandemic/post-pandemic periods, as shown in Table 2, we detected the following differences across the two periods.

- Among clinical reasons for hospitalizations, substance intoxications were more frequently recorded in the pre-pandemic period (SR = 2.67, *p* < 0.05);
- Among the psychiatric diagnoses at discharge, we observed the prevalence of adjustment reactions (SR = 3.57, *p* < 0.05) and conduct disorder (SR = 2.08, *p* < 0.05) in the pre-pandemic period, whereas depressive disorders (SR = 3.77, *p* < 0.05) and emotional disorders (SR = 4.03, *p* < 0.05) were prevalent in the pandemic/post-pandemic period;
- The HoNOSCA score statistically significantly differed between the two periods, with a decrease in the pandemic/post-pandemic period.

### 3.2. Male and Female Adolescent Hospitalizations

The male hospitalizations totaled 109 (45.2%), involving 63 males, and the female hospitalizations were 132 (54.8%), involving 90 females, with a superimposable average age (Table 3). No statistically significant differences between the two sexes were observed regarding the therapeutic adherence, which was recorded as sufficient in the majority of cases, the hospitalization regime, which was voluntary in the majority of cases, and the duration of hospitalizations, which was 9.26 ± 12.95 days on average (Table 3). The male participants reported a higher HoNOSCA score (mean = 25.55; SD = 5.77) during hospitalization than the females (mean = 21.39; SD 6.23) in a statistically significant way (t = 5.34, *t*-test, *p* = 0.000) and a more frequent comorbid substance use compared to females (Pearson Chi2 = 11.45, *p* = 0.001) (Table 3).

**Figure 2:**
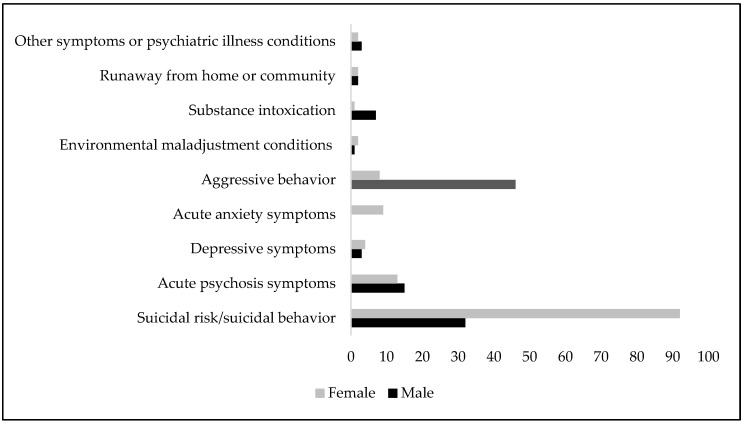
Reasons for hospitalizations in the pre- and pandemic/post-pandemic periods in males and females.

Among the male hospitalizations during the two periods of this study, aggressive behavior was the prevalent reason for hospitalizations (42.2%), with an increase in the pandemic/post-pandemic period (SR = 3.41, *p* < 0.05), whereas substance intoxication was prevalent in the pre-pandemic period (SR = 2.43, *p* < 0.05) (Pearson Chi2 = 19.76, *p* = 0.011; Fisher’s exact = 0.002) (Figure 2, Table 4).

Among the female hospitalizations during the whole study period, the risk of self-harm/suicidal behavior (69.9%) was the most frequent cause of hospitalizations, whereas acute anxiety symptoms were the statistically significant prevalent reason in the pre-pandemic period (Pearson Chi2 = 15.22, *p* = 0.055; Fisher’s exact = 0.028) (Figure 2, Table 5).

**Figure 3:**
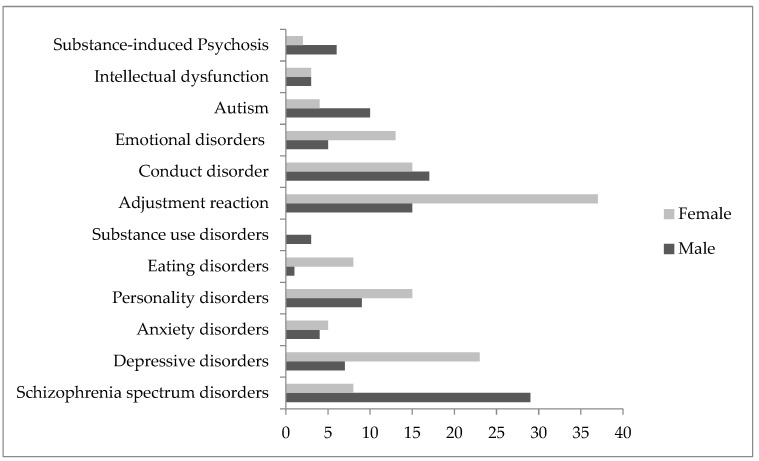
Discharge diagnoses in the pre- and pandemic/post-pandemic periods in males and females.

Among the discharge diagnoses, schizophrenia spectrum disorder (26.6%) was prevalent in males (Figure 3), with a statistically significant prevalence in the pandemic/post-pandemic period (SR = 4.03, *p* < 0.05), as well as emotional disorders (SR = 2.58, *p* < 0.05), whereas adjustment reactions were the most prevalent in the pre-pandemic period (SR = 3.14, *p* < 0.05) (Pearson Chi2 = 39.26, *p* = 0.000; Fisher’s exact = 0.000) (Table 4).

Among the female hospitalizations, the most frequent discharge diagnosis in the whole period was adjustment reactions (27.8%) (Figure 3), with a statistically significant prevalence of depressive disorders (SR = 3.07, *p* < 0.05) and emotional disorders (SR = 2.90, *p* < 0.05) in the pandemic/post-pandemic period (Table 5).

### 3.3. Seasonality of Hospitalizations

**Figure 4:**
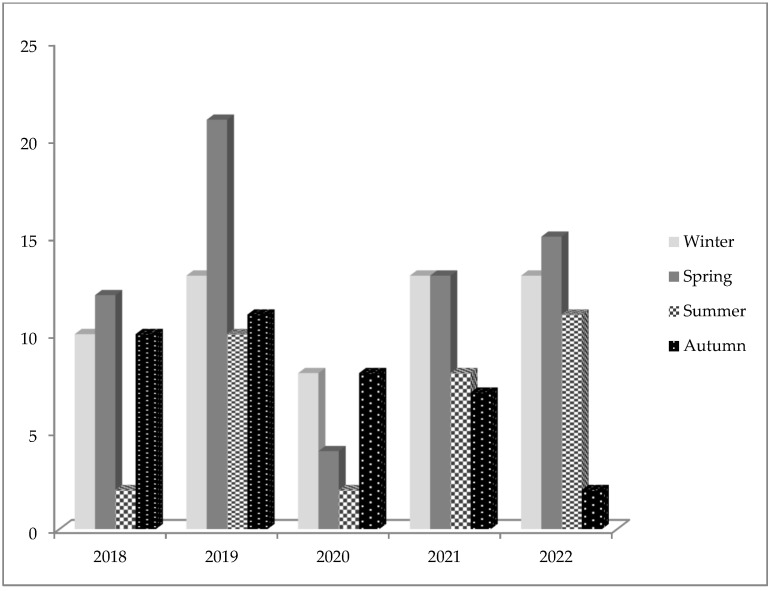
Seasonal trend in adolescent psychiatric hospitalizations from 1 January 2018 to 31 December 2022.

As shown in Figure 4, hospitalizations of adolescents increased in spring in all years but 2020. The adolescents in our sample were more frequently hospitalized in spring for suicidal risk/suicidal behavior (34.1%). No statistically significant different seasonal trend was observed in males and females (Pearson Chi2 = 3.6209, *p* = 0.305).

### 3.4. Multivariate Linear Regression

Two multiple stepwise, forward, and backward linear regression models were applied. The first one was between the lengths of hospitalization, as a dependent variable, and the other selected variables, which highlighted that the days of hospitalization, are inversely correlated with the number of previous hospitalizations in a statistically significant way (Coeff = −4.61, 95% Int. Conf. −7.91; −1.31; *p* = 0.006).

The second stepwise, forward, and backward model, between the HoNOSCA score, as a dependent variable, and the other selected variables, underscored a positive correlation with “no previous psychiatric treatments” (Coeff. = 2.25, 95% Conf. Int. −0.25; 4.25; *p* = 0.028) and negative correlations with the following variables:

- The pandemic/post-pandemic period (Coeff. = −2.50, 95% Conf. Int. −4.09; −0.91; *p* = 0.002);
- Period of previous psychiatric treatments (Coeff. = −0.39, 95% Conf. Int. 0.18; 0.60; *p* = 0.000);
- Female sex (Coeff. = −2.77, 95% Conf. Int. −4.33; −1.21; *p* = 0.00).

## 4. Discussion

This study is focused on the hospitalizations of adolescents (12–17 years) in an acute psychiatric ward during a 6-year period (from 1 July 2017 to 30 June 2023), with the aim of detecting and measuring any significant differences between the pre-pandemic and the pandemic/post-pandemic periods in the two sexes. Our analysis highlighted a decrease in adolescent hospitalizations in 2020, coinciding with the lockdown between the previous pre-pandemic period and the period following the SARS-CoV-2 outbreak. A general increase in the number of hospitalizations in the pandemic and in the following period has been confirmed by our study, with a sort of shifting between the two sexes: male adolescent hospitalizations were prevalent during the pre-pandemic, whereas, during and after the pandemic, female adolescent hospitalizations were significantly higher. This difference could be interpreted as a different vulnerability to environmental factors, as previously reported in the same setting [26]. In fact, females, who required more hospitalizations for the risk of self-harm/suicidal behavior in the whole study period, were probably more vulnerable to the stress factors during the pandemic and post-pandemic period, showing more depressive and emotional disorders as discharge diagnoses. Nevertheless, females suffered from less severe symptoms than males, as highlighted by a reduced HoNOSCA score, a result also supported by our regression model.

The reduction in hospitalizations during lockdown can be explained by the fear of infection, which may have led adolescents not to ask for help and not to contact neuropsychiatry services or the emergency services [27]. In the period following the SARS-CoV-2 outbreak, the general population reported symptoms such as anxiety, depression, and insomnia in the context of generalized fear of COVID-19, in particular by female adolescents [28], with an increase in hospitalizations, as our study highlights. The COVID-19 pandemic had a significant impact on how mental health care was delivered, on service access and on quality of care. In the first months of 2020, an overall decrease in psychiatric visits and hospitalizations was observed compared to the same period of the previous year, probably due to the underestimation of psychological needs compared to health priorities linked to the pandemic [29].

Our study highlights an increase in hospitalizations after the COVID-19 outbreak among adolescents who had already been treated in psychiatric services, confirming the observation of other authors who highlighted that the high stress of pandemic conditions, in addition to giving rise to new mental problems, has negatively affected children with pre-existing problems and health or family vulnerabilities [6,30]. This increase can be interpreted as the effect caused by the reduction in non-urgent health services and psychotherapeutic interventions in outpatient services, and possibly accumulated unmet needs during the pandemic [31]. The reduction in these services may have led to the worsening of many psychiatric pathologies, with an increase in clinical needs, which were fully satisfied again after the pandemic. On the other hand, another study reported that, during the pandemic, a greater number of adolescents not previously treated in mental health services required a first psychiatric hospitalization [32], data which can be interpreted as a different ease of access to services in different countries.

From a neurological point of view, the reduction in social contact and the increase in psychological distress in adolescence could have altered the regular neurodevelopment of the brain regions that undergo changes in the early stages of life, such as those that control the cognitive processes and also emotional regulation [33]. The interruption of a regular social life and the fear of suddenly losing loved ones have been highlighted as critical social and environmental factors [6]. Social isolation and loneliness increased the risk of depression and anxiety, and, in particular a long-lasting loneliness [4]. During the pandemic and post-pandemic periods, we reported the following observations: the HoNOSCA score of adolescents hospitalized was lower than the pre-pandemic score, a result also supported by our regression model which suggests less serious mental problems, probably due to the prevalent presence of females, who obtained lower scores than males; the use of substances, especially cannabis, increased, in accordance with other studies [34,35], whereas substance intoxication as a reason for hospitalizations decreased, suggesting less intense but more widespread substance use among adolescents; the duration of hospitalizations during the pandemic/post-pandemic remains similar to that of the pre-pandemic period, suggesting that stressful environmental factors partially conditioned our health care organization [36]. Our regression models emphasized the role of previous psychiatric treatments in reducing the length of hospitalizations and illness severity, as well as the pandemic-related changes in hospitalizing less severe conditions.

In our study, the seasonal trend in hospitalizations showed an increase in spring in all years but 2020, probably due to the health restrictions imposed by the lockdown. Moreover, adolescents with suicidal behavior were more frequently hospitalized in spring. The seasonal pattern of adolescent hospitalizations is similar to that found in a Norwegian study, which reported a reduction in access to services in summer and an opposite increase in winter [37]. An Italian study observed a seasonality of suicides with a clear peak in spring in both males and females [38]. The difference in the various seasons could be related to a stress factor related to school, as recently observed by a US study in which an association between suicides and the school calendar was reported, as opposed to a decrease in suicides in adolescents after school closures due to COVID-19 [39].

Our results underscore the increase in suicide risk after the COVID-19 outbreak, in line with another study, which found an increase in suicidal behaviors in adolescents, especially in the female population, between 2020 and 2021, compared to the previous period, while in the male population no difference in suicide rate was found between the two periods [40].

In our sample, the most frequent reasons for psychiatric hospitalizations in females was represented by suicidal behavior, whereas in males, aggressive behavior was prevalent, suggesting two different reactions to stressful environmental conditions between males and females. Similarly, the shift in hospitalizations from males to females during the pandemic can be interpreted as different vulnerabilities to environmental stressors.

Nevertheless, both male and female adolescents in our sample presented a negative impact, with aggressive behavior towards oneself or others in the pandemic probably triggered by the sudden and total reduction in the most important resilience factors, as highlighted by a recent Norwegian study [41]: school environment, emotional well-being, and good relations with their friends. We can hypothesize, in accordance with other authors [42], that pandemic conditions counteracted the adolescents’ desire for independence from their parents and the simultaneous need for connection, contributing to worsening more unstable personalities.

Moreover, the males of our sample reported a higher rate of previous hospitalizations, longer previous treatment and higher HoNOSCA scores, and more frequent diagnosis of schizophrenia spectrum disorders, suggesting more serious illness conditions than females. The vulnerability of young people to stressors related to epidemics and natural disasters has been previously demonstrated [43]. The pandemic represented a natural condition of high environmental stress, which negatively impacted adolescents’ well-being, increasing self-harm behavior [44,45]. Other authors reported that rates of suicidal ideation and self-harm in adolescents increased in the pandemic [4,16,17]. In particular, self-harming behavior and suicidal behavior increased by 27% compared to the pre-COVID-19 period [46]. This represents a dramatically relevant problem, since suicide is the second cause of death in Italy in young people between 15 and 24 years of age [46]. Self-harm affects approximately 1 in 51 adolescents in Europe [47,48]. In general, suicidal ideation or attempted suicide are now among the most frequent causes of access to emergency CANPSs. Suicide prevention has been identified as a priority objective by the World Health Organization [48].

### Limitations and Advantages of This Study

Our study presents some limitations: the single center design, which does not permit us to generalize the results to other settings; the retrospective design, which does not allow us to make causal inferences; and incomplete data, especially regarding demographic and clinical variables due to the reliance on existing medical records, which can represent an ascertainment bias. Moreover, our results did not evaluate the efficacy of counseling online in telemedicine, partially available from the CANPS during the pandemic and post-pandemic period.

Nevertheless, our study presents some advantages concerning a long observation period and the inclusion of many variables, which can describe in detail psychiatric activity during the pre- and pandemic periods. This research provides clinical information from a real-world clinical setting on a particular historical moment, deepening our knowledge of the impact of a pandemic on the mental health of adolescents. Future research conducting additional year-over-year comparisons to look for long-term longitudinal trends may provide additional information on this topic.

## 5. Conclusions

Our study highlighted an overall increase in hospitalizations in the pandemic/post-pandemic period, with a gender-specific shift and different patterns in hospitalization for reasons and diagnoses between the two sexes, suggesting that males and females experienced distinct challenges during the pandemic. In this regard, this study is one of the first to show specific sex differences in response to the pandemic in adolescents.

In light of our results, we conclude that adolescents reported a negative impact of the pandemic, increasing aggressive and self-injurious behaviors that required psychiatric hospitalizations with a specific gender difference. Moreover, during the pandemic/post-pandemic period, emotional disorders increased, and adolescents with pre-existing mental health disorders were more affected. In particular, the female population showed the greatest vulnerability, reporting an increase in depressive disorders, whereas males more frequently presented aggressive behavior and schizophrenia spectrum disorders. In the pandemic/post-pandemic, in both males and females, emotional disorders increased. Moreover, our results highlight that males presented more serious illness conditions. Those youths who had already been treated for pre-existing mental health disorders suffered the greatest fallout from the pandemic, as an increase in hospitalizations suggests.

The COVID-19 pandemic has potentially amplified the difficulties in managing the health conditions of adolescents, but has given us the opportunity to identify the most vulnerable populations, reorganizing service delivery. Our findings underscore the critical need for primary prevention measures focused on adolescent mental health, especially in the face of disasters like the COVID-19 pandemic, since this population is one of the most vulnerable to calamities and natural disasters. In particular, our results highlight the need for targeted mental health programs or specific policy recommendations differentiated between males and females to face distinct vulnerabilities and prevent the development of many pathological mental health conditions. In particular, the seasonal variations and the specific increase in suicidal behaviors in spring highlighted by our study underscore the need for seasonal considerations in psychiatric care. Future research could provide more comprehensive explanations for environmental reactions, explore the long-term impacts of the pandemic, and evaluate the effectiveness of preventive interventions on mental health conditions.

Improvements in mental health care for easier service access, for more appropriate treatments, and for mobilizing protective and preventive factors may promote the health and well-being of adolescents in the future.

## Figures and Tables

**Table 1:** Demographic and clinical variables related to adolescents hospitalized in SPDC during the pre- and pandemic/post-pandemic periods.

Variables, *n* (%)	Pre-Pandemic Period *n* = 110 (45.6)	Pandemic/Post-Pandemic Period *n* = 131 (54.4)	Total *n* = 241	Statistical Test Probability
Sex, *n* (%)				
male	61 (56.0) *	49 (37.1)	110	Pearson Chi2 = 8.54 *p* = 0.003 * SR > 2, *p* < 0.05
female	48 (44.0)	83 (62.9) *	131	
Age (years), m ± DS	15.94 ± 1.01	15.63 ± 1.18	15.77 ± 1.14	Chi2 = 3.915 Kruskal–Wallis test, *p* = 0.0478
Previous Psychiatric Treatments, *n* (%)				
CANPS	67 (60.9)	114 (87.0) *	181 (75.1)	Pearson Chi2 = 21.21 *p* = 0.000 * SR > 2, *p* < 0.05 Fisher’s exact = 0.000
Private psychiatrist	2 (1.8)	2 (1.5)	4 (1.7)	
Other or more services	5 (4.5)	3 (2.3)	8 (3.3)	
No previous psychiatric treatment	34 (30.9) *	12 (9.2)	46 (19.1)	
PREVIOUS HOSPITALIZATIONS IN SPDC, *n* (%)				
Present	60 (55.0)	60 (45.5)	120 (49.8)	Pearson Chi2 = 1.83 *p* = 0.176
Absent	49 (45.0)	72 (54.5)	121 (50.2)	
Previous Psychiatric Treatment Period (year), m ± DS	2.95 ± 3.70	3.46 ± 4.40	3.18 ± 4.03	Chi2 = 0.113 Kruskal–Wallis test, *p* = 0.7365
Hospitalization Regime, *n* (%)				
Voluntary	107 (98.2)	129 (97.7)	236 (97.9)	Pearson Chi2 = 0.4242 *p* = 0.515
Involuntary	3 (1.8)	3 (2.7)	5 (2.1)	
Substance Use, *n* (%)				
Present	83 (76.1)	115 (87.1) *	197 (81.7)	Pearson Chi2 = 5.36 *p* = 0.005 * SR > 2, *p* < 0.05
Absent	26 (23.9) *	17 (12.9)	44 (18.3)	
Treatment Adherence, *n* (%)				
Sufficient	56 (51.4)	84 (63.6)	140 (58.1)	Pearson Chi2 = 0.21 *p* = 0.900
Insufficient	53 (48.6)	48 (36.4)	101 (41.9)	

* = SR > 2, *p* < 0.05.

**Table 2:** Clinical variables related to the psychiatric hospitalizations of adolescents in SPDC during the pre- and pandemic/post-pandemic periods.

Variables, *n* (%)	Pre-Pandemic Period *n* = 110 (45.6)	Pandemic/Post-Pandemic Period *n* = 131 (54.4)	Total *n* = 241	Statistical Test Probability
Clinical Reason for Hospitalization, *n* (%)				
Suicidal risk/suicidal behavior	53 (48.2)	71 (54.2)	124 (51.5)	Pearson Chi2 = 25.78 *p* = 0.007 Fisher’s exact = 0.002 * SR > 2, *p* < 0.05
Acute psychosis symptoms	11 (10.0)	17 (13.0)	28 (11.6)	
Depressive symptoms	1 (0.9)	5 (3.8)	6 (2.5)	
Acute anxiety symptoms	7 (6.4)	2 (1.5)	9 (3.7)	
Aggressive behavior	20 (18.2)	34 (25.0)	54 (22.4)	
Environmental maladjustment conditions	1 (0.9)	0	1 (0.4)	
Substance intoxication	8 (7.3) *	1 (0.8)	9 (3.7)	
Runaway from home or community	3 (2.7)	0	3 (1.2)	
Other symptoms or psychiatric illness conditions	6 (5.5)	1 (0.8)	7 (2.9)	
Psychiatric Diagnosis at Discharge, *n* (%)				
Schizophrenia spectrum disorders	12 (10.9)	25 (19.1)	37 (15.4)	Pearson Chi2 = 50.88 *p* = 0.000 * SR > 2, *p* < 0.05
Depressive disorders	4 (3.6)	25 (19.1) *	29 (12)	
Anxiety disorders	5 (4.5)	4 (3.1)	9 (3.7)	
Personality disorders	11 (10.0)	13 (9.9)	24 (10.0)	
Eating disorders	1 (0.9)	0	1 (04)	
Substance use disorders	5 (4.5)	6 (4.6)	11 (4.6)	
Acute reaction to stress	5 (4.5)	6 (4.6)	11 (4.6)	
Adaptation reaction	30 (27.3) *	11 (8.4)	41 (17.0)	
Conduct disorder	20 (18.2) *	12 (9.2)	32 (13.3)	
Emotional disorders	0	18 (13.7) *	18 (7.5)	
Autism	8 (7.3)	6 (4.6)	14 (5.8)	
Intellectual dysfunction	3 (2.7)	3 (2.3)	6 (2.5)	
Substance-induced Psychosis	6 (5.5)	2 (1.5)	8 (3.3)	
Hospitalizations’ Duration (days), m ± DS	10.86 ± 17.04	7.92 ± 7.90	9.26 ± 12.95	Chi2 = 0.436 Kruskal–Wallis test, *p* = 0.5092
HoNOSCA score, m ± DS	25.27 ± 5.10	21.84 ± 6.81	23.49 ± 6.28	Chi2 = 17.482 Kruskal–Wallis test, *p* = 0.0001

* = SR > 2, *p* < 0.05.

**Table 3:** Demographic and clinical variables related to adolescents hospitalized in SPDC during the study period, divided by sex.

Variables, *n* (%)	Male *n* = 109 (45.2)	Female *n* = 132 (54.8)	Total *n* = 241	Statistical Test Probability
Age (years), m ± DS	15.81 ± 1.21	15.75 ± 1.08	15.77 ± 1.14	Chi2 = 0.426 Kruskal–Wallis test, *p* = 0.5138
Hospitalization Regime, *n* (%)				
Voluntary	106 (44.9)	130 (55.1)	236	Pearson Chi2 = 0.45 *p* = 0.502
Involuntary	3 (60.0)	2 (40.0)	5	
Substance Use, *n* (%)				
Present	30 (68.2)	14 (31.8)	44	Pearson Chi2 = 11.45 *p* = 0.001
Absent	79 (40.1)	118 (59.9)	197	
Treatment Adherence, *n* (%)				
Sufficient	57 (40.7)	83 (59.3)	140	Pearson Chi2 = 2.77 *p* = 0.250
Insufficient	52 (51.5)	49 (48.5)	101	
Hospitalizations’ Duration (days), m ± DS	9.31 ± 12.22	9.22 ± 13.58	9.26 ± 12.95	Chi2 = 0.534 Kruskal–Wallis test, *p* = 0.4651
HoNOSCA score, m ± DS	25.55 ± 5.77	21.39 ± 6.23	23.27 ± 6.36	t = 5.34, *t*-test *p* = 0.0000

**Table 4:** Clinical variables related to the psychiatric hospitalizations in SPDC of male adolescents during the pre- and pandemic/post-pandemic periods.

Variables, *n* (%)	Pre-Pandemic Period *n* = 61 (56)	Pandemic/Post-Pandemic Period *n* = 48 (44)	Total *n* = 109	Statistical Test Probability
Clinical Reason for Hospitalization, *n* (%)				
Suicidal risk/suicidal behavior	22 (36.1)	10 (20.8)	32 (29.4)	Pearson Chi2 = 19.76 *p* = 0.011 * SR > 2, *p* < 0.05 Fisher’s exact = 0.002
Acute psychosis symptoms	8 (13.1)	7 (14.6)	15 (13.8)	
Depressive symptoms	1 (1.6)	2 (4.2)	3 (2.8)	
Aggressive behavior	17 (27.9)	29 (60.4) *	46 (42.2)	
Environmental maladjustment conditions	1 (1.6)	0	1 (0.9)	
Substance intoxication	7 (11.5) *	0	7 (6.4)	
Run away from home or community	2 (3.3)	0	2 (1.8)	
Other symptoms or psychiatric illness conditions	3 (4.9)	0	3 (2.8)	
Psychiatric Diagnosis at Discharge, *n* (%)				
Schizophrenia spectrum disorders	7 (11.5)	22 (45.8) *	29 (26.6)	Pearson Chi2 = 39.26 *p* = 0.000 * SR > 2, *p* < 0.05 Fisher’s exact = 0.000
Depressive disorders	2 (3.3)	5 (10.4)	7 (6.4)	
Anxiety disorders	2 (3.2)	2 (4.2)	4 (3.7)	
Personality disorders	5 (8.2)	4 (8.3)	9 (8.3)	
Eating disorders	1 (1.6)	0	1 (0.9)	
Substance use disorders	3 (4.9)	0 (10.9)	3 (2.8)	
Adjustment reaction	14 (23) *	1 (2.1)	15 (13.8)	
Conduct disorders	13 (21.3)	4 (8.3)	17 (16.6)	
Emotional disorders	0	5 (10.4) *	5 (4.6)	
Autism	6 (9.8)	4 (8.3)	10 (9.2)	
Intellective dysfunction	3 (4.9)	0	3 (2.8)	
Substance-induced psychosis	5 (8.2)	1 (2.1)	6 (5.5)	

* = SR > 2, *p* < 0.05.

**Table 5:** Clinical variables related to the psychiatric hospitalizations in the SPDC of female adolescents during the pre- and pandemic/post-pandemic periods.

Variables, *n* (%)	Pre-Pandemic Period *n* = 49 (36.8)	Pandemic/Post-Pandemic Period *n* = 84 (63.2)	Total *n* = 133	Statistical Test Probability
Clinical Reason for Hospitalization, *n* (%)				
Suicidal risk/suicidal behavior	31 (63.3)	62 (73.8)	92 (69.9)	Pearson Chi2 = 15.22 *p* = 0.055 * SR > 2, *p* < 0.05 Fisher’s exact = 0.028
Acute psychosis symptoms	3 (6.1)	10 (11.9)	13 (9.8)	
Depressive symptoms	0	3 (3.6)	3 (2.3)	
Acute anxiety symptoms	7 (14.3) *	2 (2.4)	9 (6.8)	
Aggressive behavior	3 (6.1)	5 (6.0)	8 (6.0)	
Environmental maladjustment conditions	1 (2.0)	1 (1.2)	2 (1.5)	
Substance intoxication	1 (2.0)	0	1 (0.8)	
Runaway from home or community	2 (4.1)	0	2 (1.5)	
Other symptoms or psychiatric illness conditions	1 (2.0)	1 (1.2)	2 (1.5)	
Psychiatric diagnosis At discharge, *n* (%)				
Schizophrenia spectrum disorders	5 (10.2)	3 (3.6)	8 (6.0)	Pearson Chi2 = 28.50 *p* = 0.001 * SR > 2, *p* < 0.05 Fisher’s exact = 0.000
Depressive disorders	2 (4.1)	21 (25.0) *	23 (17.3)	
Anxiety disorders	3 (6.1)	2 (2.4)	5 (3.8)	
Personality disorders	6 (12.2)	9 (10.7)	15 (11.3)	
Eating disorders	2 (4.1)	6 (7.1)	8 (6.0)	
Adjustment reaction	21 (42.9) *	16 (19.0)	37 (27.8)	
Conduct disorders	7 (14.3)	8 (9.5)	15 (11.3)	
Emotional disorders	0	13 (15.5) *	13 (9.8)	
Autism	2 (4.1)	2 (2.4)	4 (3.0)	
Intellective dysfunction	0	3 (3.6)	3 (2.3)	
Substance-induced psychosis	1 (2.0)	1 (1.2)	2 (1.5)	

* = SR > 2, *p* < 0.05.

## Data Availability

All the data have been collected using the information system of the SPDC Unit, which is mandatory for all hospitalized participants. In accordance with the European GDPR 679/2016, General Authorization no. 9/2016 and Provision no. 424/2018, sensitive data cannot be exported outside of the European Union. For this reason, if requested, we may partially share our database, free of all sensitive data and any other information that may allow for the identification of study participants.
